# Subterranean Biodiversity on the Brink: Urgent Framework for Conserving the Densest Cave Region in South America

**DOI:** 10.3390/ani15192899

**Published:** 2025-10-03

**Authors:** Robson de Almeida Zampaulo, Marconi Souza-Silva, Rodrigo Lopes Ferreira

**Affiliations:** 1 Observatório Espeleológico, Rua Santa Fé, Número 66/502, Caiçaras, Belo Horizonte 30770-430, MG, Brazil; rzampaulo@yahoo.com.br; 2 Centro de Estudos em Biologia Subterrânea, Departamento de Ecologia e Conservação, Instituto de Ciências Naturais, Universidade Federal de Lavras, Lavras 37200-000, MG, Brazil; marconisilva@ufla.br

**Keywords:** impacts, underground fauna, troglobites, karst, priority areas

## Abstract

Subterranean ecosystems represent some of the most unique and fragile habitats on Earth, yet they remain poorly understood and highly vulnerable to human-induced disturbances. Despite their ecological significance, these systems are rarely integrated into conservation planning, and surface-level protected areas alone are insufficient to safeguard their biodiversity. In southeastern Brazil, a karst landscape spanning approximately 1200 km^2^, recognized as the region with the highest cave density in South America (approximately 2600 caves), is under increasing pressure from urban expansion, agriculture, and mining, all of which threaten the ecological integrity of subterranean habitats. This study sought to identify caves of high conservation priority by integrating species richness of non-troglobitic invertebrates, occurrence of troglobitic species, presence of endemic troglobitic taxa, and the degree of anthropogenic impacts, using spatial algebra and polygon-based mapping approaches. Agriculture and exotic forestry plantations (54%) and mining operations (15%) were identified as the most prevalent disturbances. A total of 32 troglobitic species were recorded, occurring in 63% of the 105 surveyed caves. Notably, seven caves alone harbor 25% of the region’s known cave invertebrate diversity and encompass 50% of its cave-restricted species. The findings highlight the global significance of this spot of subterranean biodiversity and reinforce the urgent need for targeted conservation measures. Without immediate action to mitigate unsustainable land use and resource exploitation, the persistence of these highly specialized communities is at imminent risk.

## Simple Summary

Subterranean ecosystems in southeastern Brazil are both fragile and highly biodiverse, yet they face severe threats from agriculture, mining, and urban expansion. In our survey of 105 caves, we identified 32 cave-restricted species, half of them concentrated in only seven caves. These subterranean habitats represent sites of global importance and require urgent, targeted conservation measures to prevent the irreversible loss of biodiversity.

## 1. Introduction

Karst landscapes cover approximately 15 to 20% of the Earth’s land surface, providing essential services, including groundwater storage, unique biodiversity habitats, and cultural and scientific heritage [1,2,3]. These areas are critically important because they underpin a wide range of essential values and services that support both nature and human society. They provide the foundation for ecosystems, influence biodiversity, and offer vital resources, from raw materials to air and freshwater. Beyond their ecological role, these areas enrich cultural identity, inspire scientific discovery, and provide opportunities for education, recreation, and tourism [4]. Most importantly, the geodiversity inherent to these systems drives key ecosystem services, such as soil formation, climate regulation, and natural hazard mitigation, making it indispensable for sustaining life and promoting long-term environmental resilience [5].

Despite their critical importance, karst systems are highly susceptible to environmental degradation owing to their high porosity, which allows rapid infiltration of surface pollutants and other contaminants into subsurface environments [6,7,8]. This inherent fragility is further compounded by socioeconomic pressures, particularly urban expansion and intensive carbonate mining [9]. Karst landscapes hold substantial economic value, as limestone and related carbonates rocks represent essential raw materials for the cement and construction industries, rendering these regions prime targets for extraction [9]. Mining activities are frequently concentrated near urban areas, where economic incentives and resource demand are greatest, thereby intensifying stress on these already fragile systems [10]. Such operations can trigger sinkhole formation, groundwater depletion, and severe landscape alteration, with cascading consequences for subterranean biodiversity and water security [7,10].

Although subterranean ecosystems are increasingly threatened by human activities, the current network of surface-protected areas remains inadequate for their effective conservation [11,12,13]. This shortfall arises from the inherent complexity of subterranean environments and the limited understanding of their ecological relevance. Establishing protected areas specifically aimed at safeguarding subterranean habitats is particularly challenging due to their inaccessibility and the need for specialized expertise to identify and manage them effectively [14,15,16]. Pressures from mining, pollution, urban expansion, and climate change further exacerbate their vulnerability [17,18].

A collective effort is essential to preserve subterranean biodiversity through specific policies and management strategies. Conventional approaches to mapping are complicated by the uncertain and often diffuse boundaries of subterranean habitats [15], while the rarity and endemism of many cave-dwelling species demand highly specialized taxonomic expertise for accurate identification [15,19]. The global shortage of trained taxonomists further delays the acquisition of essential biodiversity data [15,20]. Moreover, the conservation of subterranean ecosystems requires cooperation among diverse stakeholders, whose interests may often conflict. Together, these challenges highlight the urgency of adopting a coordinated and multidisciplinary approach to ensure the protection of these unique and fragile habitats [15,21].

Defining effective conservation strategies for fragile ecosystems is inherently complex, as their persistence depends on highly specific environmental conditions, such as water permeability and climatic stability. The distinctive biodiversity harbored within these systems further complicates conservation efforts, since many species display narrow habitat requirements and intricate ecological interactions [15,21]. Consequently, understanding subterranean biodiversity patterns demands a nuanced approach that considers both ecological processes and species-specific traits [22,23].

In response to these challenges, several countries have undertaken initiatives aimed at advancing knowledge of subterranean biodiversity and identifying priority areas for conservation. These efforts include long-term monitoring and targeted research programs that have revealed the complexity of subterranean life and its ecological significance [24].

In Brazil, karst landscapes cover roughly 3% of the national territory (ca. 260,000 km^2^). Over the past two decades, advances in speleological research have highlighted the role of non-carbonate formations, such as siliciclastic rocks and iron ore deposits, in speleogenesis. This broader understanding has led to revised estimates indicating that nearly 8% of Brazil’s land area (approximately 600,000 km^2^) possesses geological conditions favorable for cave formation [25,26]. Currently, around 29,000 caves are officially registered in the country [27]. However, approximately 75% of these caves are located in municipalities engaged in mining activities, while only 12% fall within legally protected conservation units, underscoring the significant tension between biodiversity protection and economic exploitation [25,28].

The state of Minas Gerais holds the largest number of known caves, with approximately 14,000 occurrences [27]. Within this state, the karstic region of Arcos, Pains, and Doresópolis (APD) stands out as the area with the highest cave density in South America, containing about 2600 registered caves within an area of just 1200 km^2^ [29]. At the same time, APD is also one of Brazil’s most important centers of limestone extraction [30]. For decades, mining has profoundly transformed this karst landscape, causing irreversible environmental impacts and leading to the destruction of countless natural cavities. In addition to mining, large portions of the landscape have been modified by agriculture and exotic forestry plantations (e.g., *Eucalyptus* spp.) [30,31].

Against this backdrop, the present study aims to develop a framework for identifying conservation-relevant sites within the APD region. By integrating measures of intrinsic ecosystem vulnerability with the intensity of anthropogenic pressures, our approach seeks to evaluate biodiversity loss risk in South America’s most cave-rich karst area, an ecosystem of global significance that is simultaneously one of the most threatened karst landscapes in Brazil. The caves are situated within the remaining areas of the Brazilian Savannah (Cerrado Biome), which is recognized as one of the most critical hotspots for global biodiversity conservation [32]. Additionally, it represents a significant protection gap within Brazil’s conservation unit system.

## 2. Materials and Methods

### 2.1. Study Area

**Figure 1:**
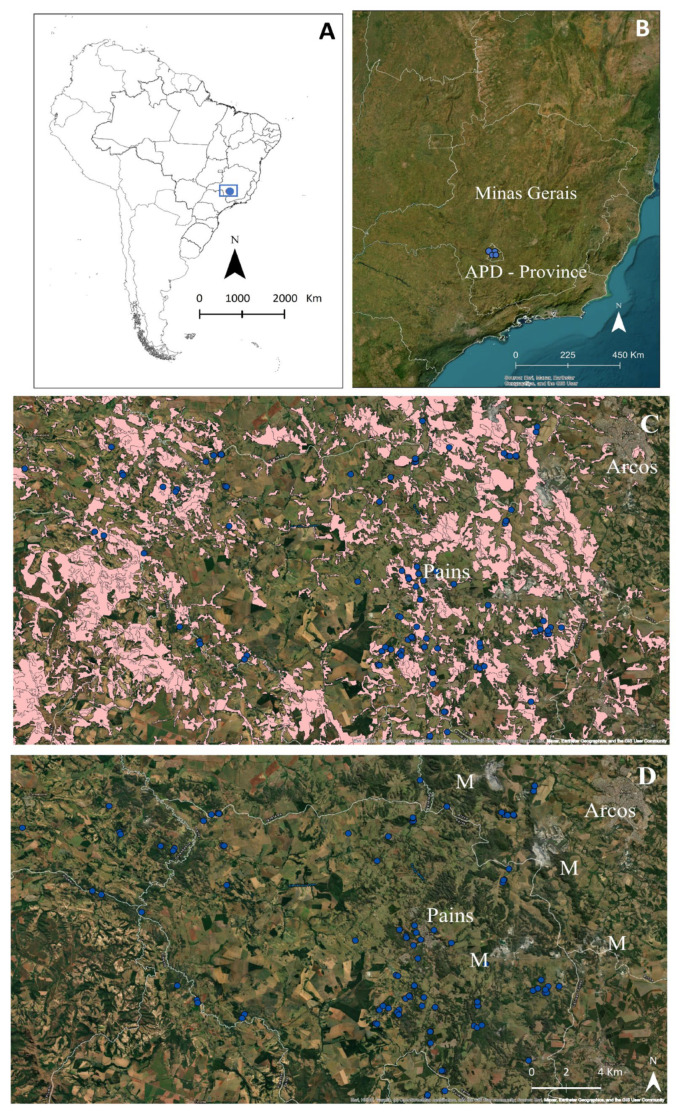
The study area is located in Minas Gerais state, Brazil (blue rectangle in (**A**) and also the caves as blue dots in (**B**)). Cave distribution in the landscape is represented by blue dots (**C**,**D**). The level of fragmentation of the natural vegetation is highlighted in magenta color (**C**)—source: https://terrabrasilis.dpi.inpe.br/downloads/ (accessed on 13 July 2025). Urban areas (Pains and Arcos) and mining activities (M) are highlighted (**D**).

The karstic area of Arcos, Pains, and Doresópolis (APD) is located in the center-west of Minas Gerais state (Figure 1A,B), within the southern portion of the Bambuí carbonate group, the largest in South America. This group is located in the southern portion of the São Francisco craton, accounting for approximately 31.5% of the total caves known in Brazil [27].

**Figure 2:**
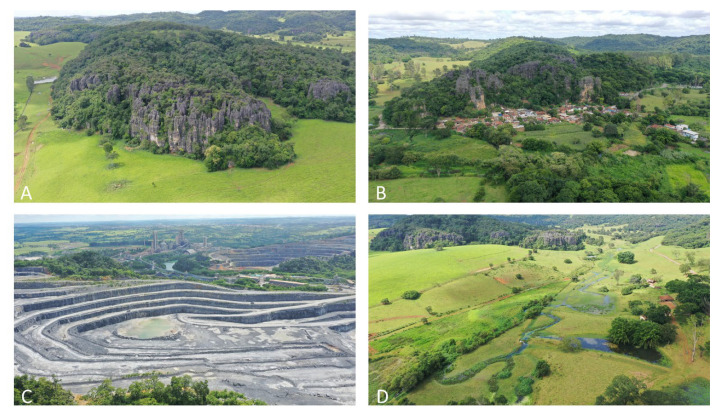
Landscape features at the municipalities of Pains, Arcos, and Doresópolis, with sparse forests on the limestone hilltops (**A**), urbanization (**B**), mining activities (**C**), and pasture (**D**).

The municipalities of Arcos, Pains, and Doresópolis (APD) are characterized by fragmented patches of vegetation and small remnants of their original landscapes [33]. Historically, this region was home to expansive savannas and seasonal deciduous forests that are part of the Atlantic Forest biome (http://mapas.sosma.org.br/ (accessed on 13 July 2025)). However, these areas have been profoundly altered due to extensive human activities, particularly mining, agriculture, and urban expansion (Figure 1C,D). In the current landscape, remnants of sparse savannahs and riparian forests can still be found along waterways and on the elevated hilltops of limestone massifs (Figure 2). Original coverage of the Atlantic Forest corresponded to 63%, 42%, and 69% of the total area of the municipalities of Pains, Arcos, and Doresópolis, respectively. The remains were 11%, 12%, and 6% from 2015 to 2016 for the municipalities of Pains, Arcos, and Doresópolis (APD), respectively (http://mapas.sosma.org.br/ (accessed on 13 July 2025)). Mining has emerged as the dominant economic activity in this region, primarily focusing on extracting limestone [34,35], featuring small cavities with an average length of 102.7 m.

### 2.2. Sampling Design

Cave selection for the inventory was based on geographical coordinates, along with criteria such as cave extension and proximity to mining operations, urban areas, agricultural lands, grazing areas, and forestry plantations. A total of 105 caves were inventoried, representing approximately 5% of the known caves within the study area (Table S1).

### 2.3. Inventory of the Cave Fauna

Invertebrates were sampled through direct intuitive searches [13,36] in all potential microhabitats, including beneath logs and rocks, as well as in organic matter accumulations such as guano deposits. Due to the structural differences in the floor between the sampling areas and the caves, the time spent searching varied among each sampling unit [36]. Collected specimens were preserved in containers with 70% ethanol for subsequent sorting and morphotype identification [36]. Sampling was consistently performed by a team of five biologists with expertise in caving and invertebrate collection, in accordance with established recommendations [36].

Voucher specimens of both troglobiont and non-troglobiont species were deposited in the Subterranean Invertebrate Collection of Lavras (ISLA), part of the Center for Studies in Subterranean Biology (CEBS) at the Federal University of Lavras, Minas Gerais, Brazil (https://www.biologiasubterranea.com.br/en/ (accessed on 28 august 2025)).

### 2.4. Determining Potential “Stygobionts” and “Troglobionts”

Potential stygobiont and troglobiont species were identified based on the presence of troglomorphic traits, which are indicative of long-term isolation and evolutionary adaptation to subterranean environments. Stygobionts (aquatic) and troglobionts (terrestrial) are species restricted to caves and/or shallow subterranean habitats, with no viable permanent populations in epigean environments. Common troglomorphic features include the reduction or loss of eyes and pigmentation, along with the elongation of sensory and locomotor appendages [36]. Many subterranean species exhibit these adaptations, often accompanied by increased body size and the development of additional sensory structures [36]. Nevertheless, some troglobionts may display limited or no apparent troglomorphy, influenced by factors such as habitat volume, light penetration in twilight zones, genetic variability, or other ecological and evolutionary processes. This variability has led to the recognition of eutroglophiles, species closely associated with subterranean habitats but not exhibiting pronounced troglomorphic traits [37]. For this reason, certain taxa in the present study are referred to as potential troglobionts, maintaining terminological consistency until more detailed ecological and genetic analyses can clarify their status.

### 2.5. Data Analysis

Priority areas for conservation were identified using the “map algebra method”, a spatial analysis technique in GIS that generates new geographic data layers through mathematical, logical, or Boolean operations applied to existing raster or grid-based datasets [12,38]. For this study, a grid of 30 × 40 polygons (totaling 1200 polygons), each measuring 10,000 m^2^, was created to cover the 1200 km^2^ study area. Caves within each polygon were evaluated based on biodiversity attributes (richness of non-troglobitic and troglobitic species, and the occurrence of stenoendemics), vulnerability, and the types of anthropogenic impacts, in order to define conservation priorities (Table 1).

The conservation value of each polygon was determined from the attributes of the caves contained within it, based on invertebrate biodiversity criteria. Each cave was classified into one of four categories (low, medium, high, and extreme) according to these attributes, and a corresponding score was assigned (Table 2). In cases where multiple caves occurred within the same polygon, the highest attribute score was considered. This procedure both emphasized caves with the greatest conservation relevance and increased the weight of polygons containing a higher number of caves. Finally, attribute layers were combined using the map algebra method, in which the sum of scores from each attribute produced a final conservation value for each polygon [38].

The priority classes for conservation were defined using the “Natural Breaks” classification method, which categorizes interval/ratio data based on a subjective recognition of gaps in the data distribution [42]. This approach minimizes within-class variance while maximizing variance between classes, thereby enhancing the interpretability of spatial patterns [43]. To visualize the outcomes of these analyses, thematic maps were generated for each attribute considered, along with a composite map representing the overlap of attributes, thereby delineating priority areas for conservation (Figures S1–S5).

A linear regression was performed to detect relationships between cave extension and non-troglobite species richness [44]. Since only one sample was taken from each cave, we tested whether the dry and rainy periods affected the number of species by using the richness of non-troglobite and troglobite species obtained for the caves, which were grouped according to the season in which the caves were sampled [44]. Such groups were then compared through the Kruskal–Wallis non-parametric test [45]. These periods (dry and wet seasons) were defined based on rainfall and soil water availability values obtained from a weather station located approximately 60 km from the study area [46]. The rainy season in the region occurs between October and March (*n* = 54 caves), while the dry season occurs between April and September (*n* = 49 caves). The Jackknife 1 estimator was performed to achieve the level of ‘completeness’ of the sampling effort.

## 3. Results

The most frequent environmental alteration observed among the five recorded impact types was the replacement of natural vegetation with pastures. Agriculture and forestry activities, particularly plantations of *Eucalyptus* spp., accounted for the largest share of impacts (54%), followed by mining (15%), urbanization (7%), and paved roads (6%).

In total, 63,651 invertebrates were accounted, representing 1313 species distributed across 51 orders and 226 families. The mean richness of non-troglobitic species was 49.2 species per cave (SD = 17.1), with values ranging from 17 to 93 species per cave (Figure S1). Based on richness categories, 11 caves exhibited extreme richness (75–93 species), 23 were classified as high richness (56–74 species), 47 as medium richness (37–55 species), and 24 as low richness (17–36 species).

**Figure 3:**
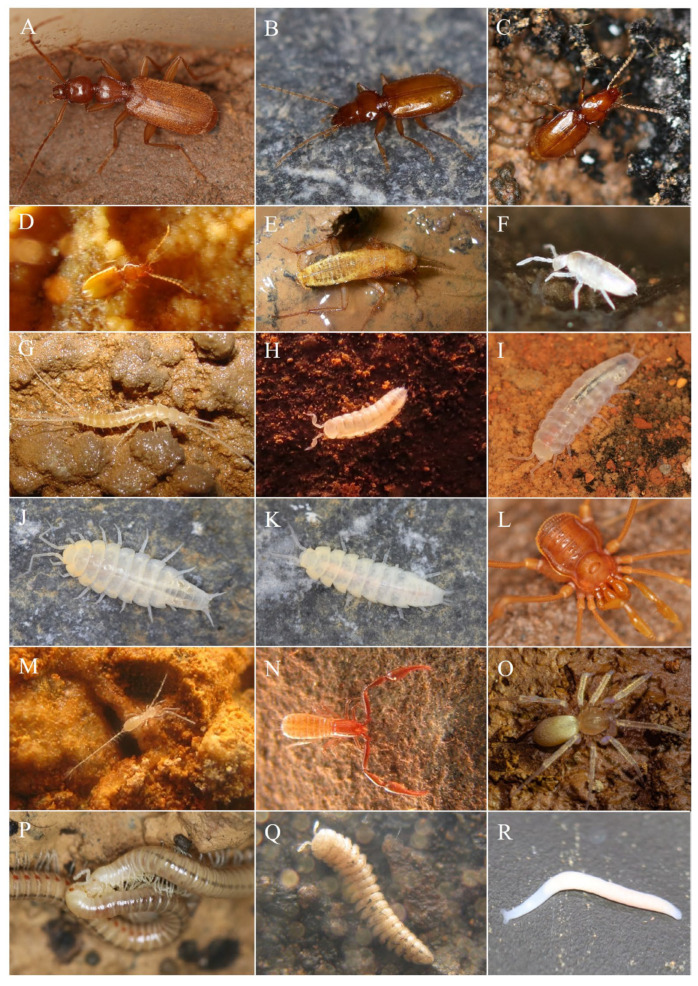
Some of the Troglobite species recorded in the karstic region of Arcos, Pains and Doresópolis: (**A**) Carabidae: *Coarazuphium pains*; (**B**) Carabidae: *Perigona* sp.; (**C**) Carabidae: *Perigona spelunca*; (**D**) Carabidae: *Paratachys* sp.; (**E**) Blattellidae: *Litoblatta* sp.; (**F**) Entomobryidae: *Cyphoderus* sp.; (**G**) Microcoryphia: Nicoletiinae sp.; (**H**) Styloniscidae: *Pectenoniscus* sp.; (**I**) Styloniscidae: *Spelunconiscus* sp.1; (**J**) Styloniscidae: *Spelunconiscus* sp.2; (**K**) Styloniscidae: *Spelunconiscus* sp.3; (**L**) Cryptogeobiidae: *Paratrichomatus infernalis*; (**M**) Eukoeneniidae: *Eukoenenia cavatica*; (**N**) Ideoroncidae; (**O**) Prodidomidae: *Lygromma* sp.; (**P**) Pseudonannolenidae: *Pseudonannolene ambuatinga*; (**Q**) Oniscodesmidae: *Crypturodesmus* sp.; (**R**) Microturbellaria.

A total of 32 species with troglomorphic features were identified (Figure 3, Table S1), occurring in 66 of the 105 sampled caves (63%). Of these, only six species (19%) have been formally described to date: *Coarazuphium pains* Álvarez & Ferreira, 2001; *Pseudonannolene ambuatinga* Iniesta & Ferreira, 2013; *Eukoenennia cavatica* Souza & Ferreira, 2016; *Metopiellus painensis* Asenjo, Ferreira & Zampaulo, 2017; *Matta nuusga* Brescovit & Cizauskas, 2019; and *Perigona spelunca* Pellegrini, Ferreira & Vieira, 2022. Troglobitic species richness ranged from 0 to 14 species per cave, with Eden Cave hosting the highest number of species (Figure S2, Table S1). The mean richness of troglobitic species was 1.5 per cave (SD = 2). Among caves with troglobitic species, 42 were classified as low richness (1–2 species), 16 as medium richness (3–4 species), seven as high richness (5–6 species), and only one as extreme richness (≥7 species) (Figure S2, Table S1). A positive relationship was observed between the number of troglobitic species and cave size (F_(1,15)_, R^2^ = 0.19; *p* < 0.01). However, no significant differences were detected in the richness of either non-troglobitic or troglobitic species between the dry and rainy seasons.

Of the 32 troglobite species found, 21 (65.6%) had a single occurrence. Such species are represented by Carabidae beetles (three species), a Blattellidae cockroach (*Litoblatta*), a palpigrade (*Eukoenennia cavatica*), two Nicoletiidae silverfish, and 14 species of Styloniscidae isopods (*Spelunconiscus* and *Pectenoniscus*). Eden and Cavalinho Caves presented the highest number of endemic troglobite species with two species each, while 18 caves presented a single endemic troglobite species each (Figure 3 and Figure S3). No cave was considered in the extreme category (three endemic troglobite species). The estimated troglobitic species richness suggests that the sampling effort achieved good levels of completeness, as the observed richness (32 spp.) corresponds to over 65% of the estimated richness.

**Figure 4:**
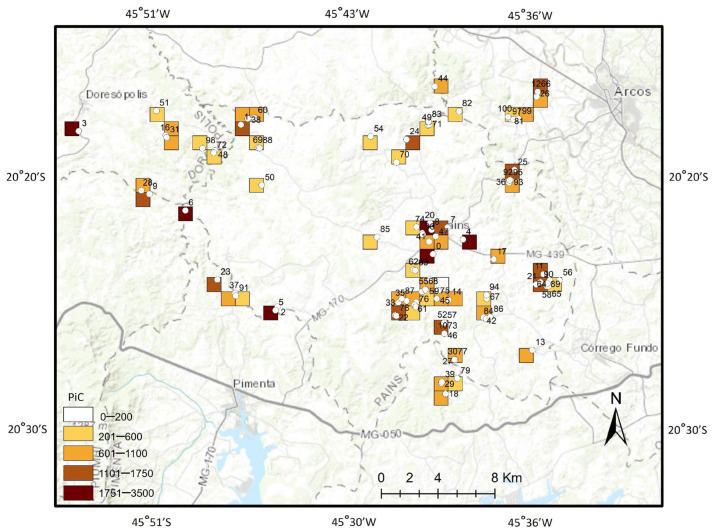
Indication of polygons as priority areas for cave invertebrate conservation (PiC) in the karst of Arcos, Pains, and Doresópolis, Minas Gerais state, Brazil (number from 201 to 3500). Details in Table S1. (0) Gruta do Éden, (1) Gruta do Cavalinho, (2) Gruta do Brega, (3) Buraco dos Curiós, (4) Gruta Serra Azul, (5) Gruta Santuário, (6) Gruta Zizinho Beraldo, (7) Gruta do Isaias, (8) Zé da Fazenda I, (9) Gruta do Barreado II, (10) Loca dos Negros II (Seca), (11) Gruta Fazenda Amargoso, (12) Gruta Cazanga, (13) Gruta Cinderela, (14) Gruta do Zé Serafim, (15) Gruta Quimvale I, (16) Gruta Helinho II, (17) Gruta do Vicente Amargoso, (18) Gruta da Paca, (19) C3 Bocaininha, (20) Gruta Paranoá, (21) Gruta das Cerâmicas, (22) Loca D’água (Sumidouro), (23) Gruta Marinheiros, (24) Gruta Olhos D’Água, (25) Gruta da Vila Corumbá, (26) Gruta Branca, (27) Gruta Água Limpa II, (28) Gruta dos Milagres, (29) Gruta Dimas II, (30) Gruta Água Limpa I, (31) Gruta do Helinho I, (32) Gruta Lenticular, (33) Gruta da Ponte Velha I, (34) Gruta do Sr. Francisco, (35) Gruta do Coelho, (36) Gruta Ninfeta de Cima, (37) Gruta do Sumidouro, (38) Gruta do Liveirinho, (39) Gruta do Dimas I, (40) Gruta do Zé Erpídio, (41) Gruta Sarmento, (42) Gruta Cristais, (43) Gruta do Físico, (44) Gruta dos Fornos I, (45) Gruta do Tio Rafa II, (46) Loca dos Negros I (Água), (47) Gruta Zé da Fazenda II, (48) Gruta da Fumaça II, (49) Gruta do Cornélio II, (50) Gruta do Capoeirão, (51) Gruta dos Coqueiros, (52) Gruta do Café, (53) Gruta do Albano, (54) Gruta do Tio Ferreira, (55) Gruta Macacos I, (56) Gruta do Grande Salão, (57) Sistema Conchas, (58) Toca Bicho Desconhecido, (59) Gruta Macacos III, (60) Gruta da Guela, (61) Gruta do Teto Alto, (62) Gruta Tamafi I, (63) Gruta Tamafi II, (64) Sistema Aranha Gigante, (65) Gruta do Paleopiso, (66) Gruta do Zé Colméia, (67) Gruta do Zé Serafim III, (68) Gruta Macacos II, (69) Gruta Terra Amarela I, (70) Gruta São Lourenço I, (71) Gruta do Cornélio I, (72) Gruta da Fumaça III, (73) Loca dos Negros III, (74) Gruta Duas Bocas, (75) Gruta do Tio Rafa I, (76) Gruta Dolina dos Angicos, (77) Gruta da Água Limpa III, (78) Loca D’água (Ressurgência), (79) Gruta da Índia, (80) C6 Bocaininha, (81) C7 Bocaininha, (82) Gruta do Zé Geraldão, (83) Gruta do Cornélio III, (84) Gruta do Mastodonte, (85) Gruta do Veado, (86) Loca Feia, (87) Gruta da Ponte Velha II, (88) Gruta Terra Amarela II, (89) Gruta do Tronco, (90) Gruta Asa de Maripopsa, (91) Gruta Sumidouro do Lixo, (92) Gruta Ninfeta de Baixo, (93) Gruta da Mineração, (94) Gruta do Zé Serafim II, (95) Gruta Ninfeta III, (96) Gruta da Manada I, (97) C1 Bocaininha, (98) Gruta Dico Ramiro, (99) C4 Bocaininha, (100) C8 Bocaininha, (101) Gruta Timboré II, (102) Gruta do Cornélio IV, (103) Gruta Timboré I, (104) Abismo da Manada II.

The vulnerability assessment indicated that all sampled caves (100%) exhibited some degree of human interference in their surrounding areas. Among the 105 evaluated caves, three were classified as having extreme vulnerability (weight = 1000; Gruta do Éden, Gruta Serra Azul, and Vila Corumbá), nine as high vulnerability (weight = 500), 59 as medium vulnerability (weight = 250), and 34 as low vulnerability (weight = 100) (Figure 4 and Figure S4; Table S1).

By overlapping the four evaluated attributes, six areas (10%), encompassing eight caves, were identified as priority sites for the conservation of cave invertebrate biodiversity within the APD karst region (Figure 4 and Figure S5; Table S1). The eight priority caves, listed in order of importance, are Eden Cave, Serra Azul Cave, Buraco dos Curiós Cave, Zizinho Beraldo Cave, Santuário Cave, Brega Cave, Paranoá Cave, and Cavalinho Cave. Collectively, these sites harbor 341 species (26% of the total richness recorded in the study area) and 16 troglobitic species, representing 50% of all troglobites documented. In addition, 11 areas (18%) were classified as high priority, 23 areas (38%) as medium priority, and 20 areas (34%) as low priority for conservation (Figure 4).

## 4. Discussion

The Brazilian cave fauna has only begun to be relatively well-documented over the past three decades. Although early studies were concentrated in a few caves, research has primarily focused on limestone systems and, more recently, on iron ore formations [35]. While approximately 29,000 caves are currently registered in Brazil, projections suggest that the country may harbor more than 310,000 caves [9]. Despite this vast potential, only about 2000 caves have been biologically surveyed, representing a mere 0.6% of the estimated total [35,47,48,49]. In this context, studies aimed at characterizing subterranean biodiversity and establishing conservation priorities are of particular importance, given the still limited knowledge of Brazil’s cave fauna [49,50,51].

Notably, the caves inventoried in this work are located within the remaining areas of the Brazilian Savannah (Cerrado Biome); this is considered one of the most critical hotspots for the conservation of global biodiversity and the most significant protection gap in the Brazilian conservation unit system [32]. Unfortunately, although the studied area represents a prominent speleological unit in Brazil, it is not currently covered by any governmental conservation unit.

In the APD karst region, despite a long history of intense environmental impacts, a high concentration of troglobitic species was recorded. Of the 32 troglomorphic species identified in this study, 26 remain undescribed. Furthermore, additional troglobitic taxa have been reported from other caves in the region not included in this survey, totalling 50 cave restricted species in the area [52]. This fact suggests that the true number of cave-restricted species is considerably higher and continues to grow. This finding reinforces the notion that Brazilian cave fauna remains significantly underestimated, reflecting a pronounced *Linnean shortfall*, and highlights the urgent need for investment in basic taxonomic research to document this biodiversity [53].

A total of 21 species were identified as stenoendemics, restricted to a single cave, and therefore classified as Critically Endangered under the International Union for Conservation of Nature (IUCN) criteria, due to their extremely small populations and highly restricted geographic ranges [54]. Investment in taxonomic description and formal recognition of these species should thus be considered an urgent priority. In several cases, both stygobionts and troglobionts exhibited extreme endemism, with distributions limited to a single cave or to a small cluster of nearby caves [36,55]. Such narrow ranges underscore their heightened vulnerability to environmental change, habitat disturbance, and other anthropogenic pressures, reinforcing the critical importance of conserving subterranean biodiversity within these fragile ecosystems [56].

Several studies examining the spatial distribution of troglobitic and stygobitic species across broad geographical scales have employed grid-based mapping techniques to standardize spatial analyses [57,58,59]. This approach enables meaningful comparisons among uniform cells, thereby improving the understanding of distribution patterns and supporting the prioritization of conservation efforts.

In the present study, the positive relationship observed between cave size, overall species richness, and the richness of cave-restricted species highlights the importance of these attributes for the conservation of subterranean fauna. Larger caves typically exhibit more stable environmental conditions and greater geomorphological complexity, which favor the importation of resources and the persistence of ecological processes [35,60,61,62,63]. These conditions can also promote the evolution and maintenance of higher numbers of troglobitic and stygobitic species. Consequently, prioritizing the conservation of larger caves with elevated invertebrate richness in the study area is likely to ensure the protection of a greater proportion of cave-restricted species.

However, it is important to note that relying solely on the richness of troglobitic species as a parameter for defining conservation priorities may be insufficient. The presence of troglobitic species, without information on their population status, does not necessarily reflect the ecological integrity of a subterranean system, particularly in caves subject to significant anthropogenic disturbance [39]. From this perspective, incorporating additional parameters (such as overall species richness, taxonomic composition, and community structure) can provide a more robust and reliable diagnosis of the conservation status of subterranean ecosystems.

### Indication of Priority Areas

According to Brazilian legislation, caves have been recognized as property of the Union since 1988 and, from 1990 onward, were granted integral protection. At that time, their use was restricted to scientific and tourist purposes under strict requirements to ensure their physical integrity and ecological balance [41]. However, a legislative amendment in 2008 significantly weakened this protection, allowing caves to be suppressed by economic enterprises, provided they were first classified by relevance (maximum, high, medium, or low). This classification is based on information from environmental studies encompassing speleological, geological, paleontological, biological, cultural, and other attributes [41].

The APD karst has experienced centuries of land use change, initially through the conversion of natural areas to agriculture and, more recently, through the intensification of mining. Over the past five decades, limestone extraction has expanded markedly, resulting in the suppression of extensive karst areas and the loss of numerous caves. The present study demonstrated that all evaluated caves exhibited impacts in their surrounding areas, including those of high biological importance and with elevated concentrations of troglobitic species. Because caves are oligotrophic environments, where most trophic inputs originate from surface landscapes, alterations to karst surfaces can directly disrupt ecosystem balance and threaten subterranean biodiversity, ultimately leading to species extinctions [39,63,64,65].

Furthermore, the mineral rights already granted by the Brazilian government across the study area allocate nearly all karst lands of the APD for potential future mining activities (Figure S6). This designation places the entire region at serious risk of degradation and large-scale cave destruction. Given these conditions, and in light of the weakened legal framework for cave protection [66], the caves and karst areas identified here as conservation priorities must be considered of critical importance for the long-term preservation of Brazilian subterranean biodiversity. In particular, the region surrounding the municipality of Pains should be treated as a priority of the highest urgency, as it harbors numerous caves while simultaneously concentrating several active mining operations. Establishing legally protected areas in such zones is therefore imperative to ensure the conservation of subterranean biodiversity within the APD region.

## 5. Conclusions

This study highlights the critical importance of the APD karst area as a reservoir of unique and highly vulnerable subterranean biodiversity. Although Brazil has more than 29,000 registered caves, only a small fraction has been biologically surveyed, reflecting a pronounced Racovitzan impediment and leaving much of the country’s subterranean biodiversity hidden within unexplored environments. The challenge of documenting, mapping, and conserving such biodiversity is immense, yet essential. The APD exemplifies this gap, containing the highest cave density in South America and supporting a remarkable concentration of cave-restricted species, while simultaneously being subjected to intense quarrying and other anthropogenic pressures. Most of these cave-restricted species remain undescribed and are confined to single caves, which, under IUCN criteria, render them Critically Endangered.

Our findings emphasize the urgent need for conservation measures that prioritize larger caves with higher species richness, as well as those hosting stenoendemic species. However, conservation planning should not rely exclusively on the presence of troglobitic species as indicators of ecosystem integrity; instead, it should integrate multiple parameters, including overall richness, taxonomic composition, and ecological functioning.

Protecting and studying the APD caves is vital not only for preserving endemic and stenoendemic species but also for advancing the broader understanding of subterranean ecosystems. Strengthening basic research, expanding biological inventories, and implementing conservation policies that reflect both the ecological and evolutionary value of these systems are fundamental steps toward their long-term preservation. Above all, it is urgent to establish conservation units in the APD karst area, safeguarding the most biologically relevant caves to ensure their physical and biological integrity. These caves are irreplaceable, and their loss would mean not only the extinction of unique species but also the disruption of critical ecological processes on which surrounding landscapes depend.

## Figures and Tables

**Table 1:** Four criteria are used to build a cave vulnerability framework and to determine priorities for cave conservation.

Criterion	Description
Non-troglobite Species Richness	Total number of non-troglobite species observed per cave. This value reflects the ecological connectivity and the cave’s contribution to the broader subterranean network [39].
Troglobite Species Richness	Number of species exclusively adapted to subterranean environments (troglobites). Represents the taxonomic and evolutionary significance of the cave habitat [12,39,40].
Endemic Troglobite Species	The number of troglobite species found in only one cave (stenoendemic) [12,40]. These species are highly endemic and extremely vulnerable to extinction due to environmental impacts, such as surface alteration or direct cave destruction (e.g., from mining activities).
Vulnerability	A 250 m buffer was established around each cave to evaluate the presence or absence of human impacts. Caves were assessed for vulnerability to anthropogenic disturbances (pollution, land use change, groundwater contamination). This buffer is based on Brazilian Decree No. 6640 (2008) [41].

**Table 2:** The caves were evaluated based on their biodiversity and vulnerability to impact. Category (CT), score (SC), non-troglobites species richness (nTS), Troglobite species richness (TbS), Endemicty (EnD), Vulnerability (VuL), Priorities for conservation (PiC).

CT	SC	nTS	TbS	EnD	VuL	PiC
Low	100	17–36	1–2	0	1	201–600
Average	250	37–55	3–4	1	2	601–110
High	500	56–74	5–6	2	3	1101–176
Extreme	1000	75–93	6–7	3	4, 5	1751–350

## Data Availability

The data supporting the findings of this study are available upon request from the corresponding author. The Editor-in-Chief has waived the required archiving due to privacy or ethical restrictions.

## Supplementary Materials

The following supporting information can be downloaded at https://www.mdpi.com/article/10.3390/ani15192899/s1: Figure S1: Classification of caves based on the non-troglobite species richness distribution at Arcos, Pains, and Doresópolis (APD) karstic area; Figure S2: Classification of caves based on the troglobite species richness distribution at Arcos, Pains, and Doresópolis (APD) karstic area; Figure S3: Classification of polygons based on the stenoendemic troglobite species richness distribution at Arcos, Pains, and Doresópolis (APD) karstic area; Figure S4: Classification of polygons based on the vulnerability criteria Arcos, Pains, and Doresópolis (APD) karstic area; Figure S5: Classification of polygons based on the priority for conservation at Arcos, Pains, and Doresópolis (APD) karstic area; Figure S6: Caves known for the karst area of Arcos, Pains, and Doresópolis and mining rights (orange polygons) granted by the Brazilian government; Table S1: List of caves sampled, their geographic location (WGS 84 Datum), and their classification in each biological attribute. Non-troglobite species (nTS), Troglobite species richness (TbS); Endemicity of Troglobite species (EnD), Vulnerability (VuL), and Conservation priority (PiC).
