# Supplementary figures and images for: Subterranean Biodiversity on the Brink: Urgent Framework for Conserving the Densest Cave Region in South America
Source: Animals (Basel). 2025 Oct 3;15(19):2899. doi: 10.3390/ani15192899 (PMC12523527; doi:10.3390/ani15192899)

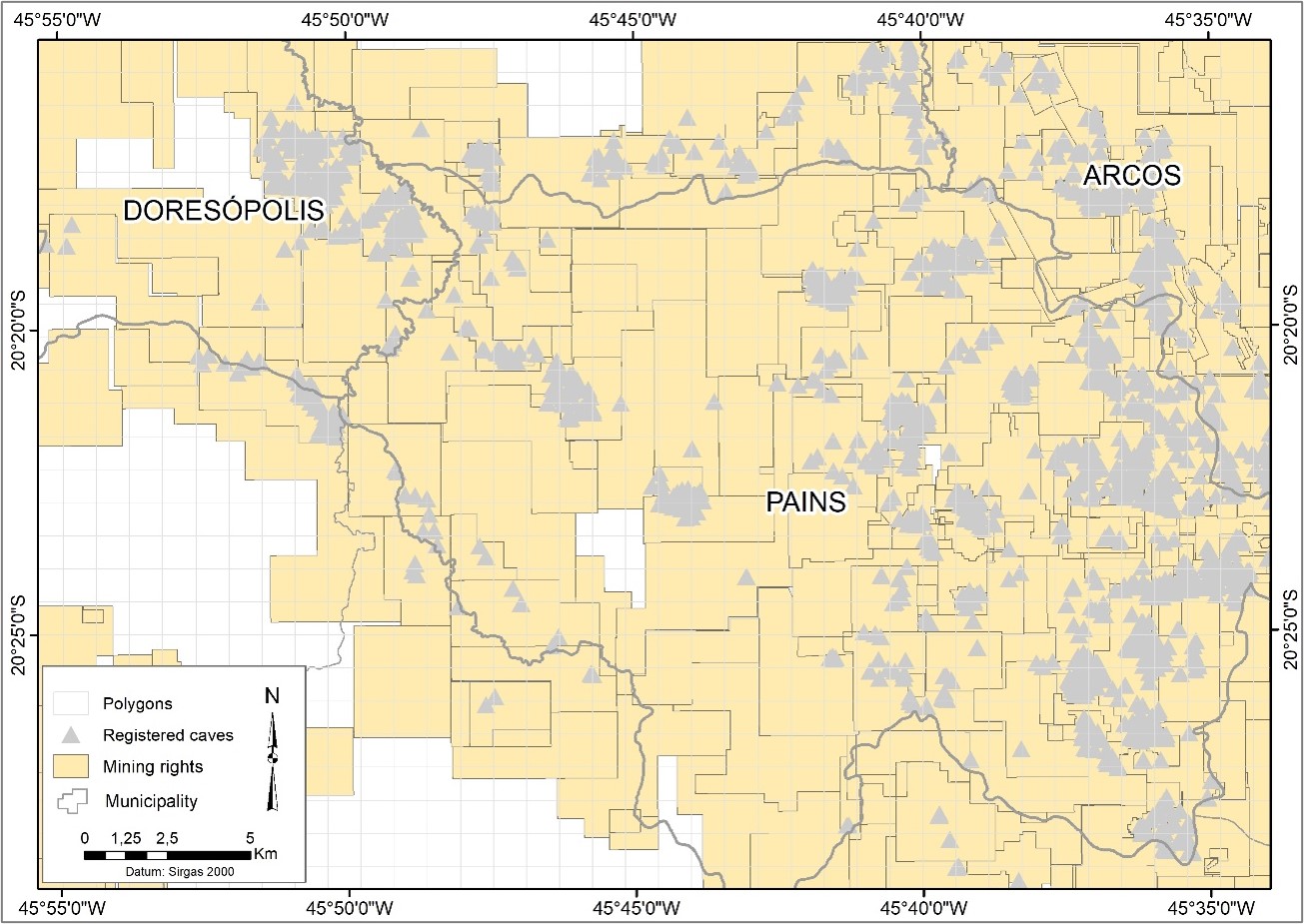

Supplement: Supplementary file 1 [file animals-15-02899-s001.zip › Figure S6.jpg]
